# Simulation of epiretinal prostheses - Evaluation of geometrical factors affecting stimulation thresholds

**DOI:** 10.1186/1743-0003-8-44

**Published:** 2011-08-19

**Authors:** Harsha Kasi, Willyan Hasenkamp, Gregoire Cosendai, Arnaud Bertsch, Philippe Renaud

**Affiliations:** 1 Microsystems Laboratory (LMIS4), Ecole Polytechnique Fédérale de Lausanne (EPFL), Lausanne 1015, Switzerland; 2 Second Sight® Medical Products, Inc., Sylmar, CA 91342, USA

## Abstract

**Background:**

An accurate understanding of the electrical interaction between retinal prostheses and retinal tissue is important to design effective devices. Previous studies have used modelling approaches to simulate electric fields generated by epiretinal prostheses in saline and to simulate retinal ganglion cell (RGC) activation using passive or/and active biophysical models of the retina. These models have limited scope for studying an implanted human retinal prosthesis as they often do not account for real geometry and composition of the prosthesis-retina interface. This interface consists of real dimensions and location of stimulation and ground electrodes that are separated by the retinal tissue and surrounded by physiological fluids.

**Methods:**

In this study, we combined the prosthesis-retina interface elements into a framework to evaluate the geometrical factors affecting stimulation thresholds for epiretinal prostheses used in clinical human trials, as described by Balthasar *et al*. in their Investigative Ophthalmology and Visual Science (IOVS) paper published in 2008 using the Argus I epiretinal implants. Finite element method (FEM) based computations were used to estimate threshold currents based on a threshold criterion employing a passive electric model of the retina.

**Results:**

Threshold currents and impedances were estimated for different electrode-retina distances. The profiles and the values for thresholds and impedances obtained from our simulation framework are within the range of measured values in the only elaborate published clinical trial until now using Argus I epiretinal implants. An estimation of resolution for the electrodes used in these trials was provided. Our results reiterate the importance of close proximity between electrodes and retina for safe and efficient retinal stimulation.

**Conclusions:**

The validation of our simulation framework being relevant for epiretinal prosthesis research is derived from the good agreement of the computed trends and values of the current study with measurements demonstrated in existing clinical trials on humans (Argus I). The proposed simulation framework could be used to generate the relationship between threshold and impedance for any electrode geometry and consequently be an effective tool for design engineers, surgeons and electrophysiologists.

## Background

More than 40 million people around the world suffer vision impairment due to retinal degeneration diseases e.g. retinitis pigmentosa (RP) and age-related macular degeneration (AMD) [1]. These diseases are incurable by current treatments [2] and affect the retinal photoreceptor cells that stop functioning and eventually die. Electronic prosthetic devices [3] can be implanted to replace the functionality of the photoreceptors by exciting the secondary neurons of the retina leading to a partial perception of the visual scenario. Many groups (refer to the review [3]) worldwide are working on different devices based on the placement of the implant with respect to the retina. One such device is the epiretinal implant, which target retinal ganglion cells (RGCs) by having the electrodes facing the inner surface of the retina. Several modelling and simulation studies on retinal prostheses [4-11] have been performed to analyse the bioelectronic interface between the retina and the electrodes, but not yet in an integrated framework. To solve this issue, preliminary steps in constructing a complete framework for simulating epiretinal prosthesis have been developed in the present study to evaluate the factors affecting the activation thresholds of RGCs. The framework described here is similar to the one reported recently by us to study spatial extent of stimulation and effect of electrode-tissue gap in subretinal implants [12]. Throughout this manuscript, the word *activation *can mean activation of one or more RGCs as a result of extracellular stimulation.

Two major electrical parameters responsible for affecting the efficiency of retinal prostheses [13] are: (i) the fluctuation of current amplitude for activation (threshold current) that can occur due to unstable positioning of the electrode array on the inner retinal surface, electrochemical alterations in the electrodes, or neurophysiological remodelling of the retina. (ii) The charge density necessary to elicit visual percepts to permit long-term stimulation without damaging the retina or the electrodes. The determination of threshold current and charge density is important for achieving safe stimulation. Appropriately, electrode-retina distances along with the electrode geometry are factors influencing the retinal stimulation. The development of an integrated simulation framework can predict the stimulation parameters by including these factors in the model.

An electrode-retina distance contributes to the varying current spread from the electrodes causing changes in the stimulation area in the retina and therefore affects the resolution of the prosthesis. In vitro electrophysiological data and analytical calculations suggest that the threshold currents rise rapidly with increasing distance of the electrodes from the retinal surface [14,15].

Electrode geometry has an effect on the current required for RGC activation. In vitro experiments [16] have established that the threshold current necessary to elicit spikes in RGCs has a power law relationship with electrode area. Incorporating these geometrical factors affecting perceptual thresholds in a simulation framework can be of interest to: design engineers of retinal implants-aiding them to determine optimal electrode schemes for retinal stimulation by predicting values for spatial extents (resolution) and probable electrochemical effects on the electrode surface; surgeons - assisting them after surgery to verify the distance between implant and the retina in addition to a visual confirmation [17]; and electrophysiologists - to estimate the threshold current, voltage or charge needed during an actual stimulation trial [13].

Presently, proximity of the retina to the electrodes is verified by two different techniques after implantation of retinal prostheses. Optical coherence tomography (OCT) is one of the methods that reveal only proximity of the edges of the device to the retina for a non-transparent retinal implant. The other technique, known as impedance analysis [18] uses the changes in impedance to estimate the electrode-retina distance. The changes in impedance occur when the implant moves closer or away from the retina. The utilisation of an integrated framework can predict the impedance associated with an electrode-retina distance considering different electrode geometries.

Discretisation methods such as Finite Element Method (FEM) can be employed to compute electric fields within the retina for different electrode geometries and electrode-retina distances in either epiretinal or subretinal schemes. For real geometries, it is cumbersome to analytically calculate and predict the effect of these factors on retinal stimulation. To resolve such a complex electrostatic problem, FEM can be used in a simulation framework. For a successful simulation, the framework should include anatomically correct retina model describing electrical characteristics of the retinal layers [19] with due attention to the retina size corresponding to an actual implantation scenario. In addition, the framework should incorporate models for the stimulation and ground electrodes, and the physiological fluid.

For our studies, a simulation framework was built integrating the prosthesis-retina interface elements involved in an epiretinal prosthesis closely resembling the one used in the framework of the only and most comprehensive published human trials until now using Argus I epiretinal implants by de Balthasar *et al*. [13]. Following are the features of our framework that has not been dealt by previous modelling studies on epiretinal stimulation: (i) the location and dimensions of stimulation and ground electrodes were adapted to a real implantation scenario; (ii) a realistic representation of the electrical properties of the retina; (iii) choice of a simplified, yet realistic activation threshold criterion based on a recent analytical study [20] that incorporates the critical stimulation parameters such as stimulus type (monophasic/biphasic), shape (cathodic/anodic) and duration under a single unified model (iv) Predictions on threshold currents and impedance with varying electrode-retina distances for different electrode dimensions. Using this framework, variation of threshold currents and impedances were computed using different electrode-retina distances and disc electrode sizes. In order to demonstrate the relevance of our simulation framework to implanted human epiretinal prosthesis, the frame of reference for the computed results from our simulation framework is the most recent experimental data on geometrical factors affecting perceptual thresholds presented in Argus I trials. We estimated lateral extents of stimulation for the electrodes, which provides an indication to the resolution of the epiretinal prosthesis used in those trials. Subsequently, this simulation model can be easily modified to predict the efficiency of novel electrode geometries for epiretinal prostheses.

## Methods

### Simulation model

Elements of the framework are: physiological fluid, stimulation and return (ground) electrodes, and retina. Physiological medium encompasses the implant until the photoreceptor layer of the retina and defined to have a resistivity of 2 Ω·m. The retinal pigment epithelium (RPE) is represented by a highly resistive block above the retina, which is set to a resistivity of 500 Ω·m. The stimulation electrode is positioned on the RGC layer side of the retina in the form of a planar disc embedded in an insulated substrate and located at the geometrical centre of the entire model geometry. The insulation (flexible sheet of the implant) was defined with a resistivity of 1 × 10^17 ^Ω·m [21] corresponding to Polyimide and an electrode resistivity of 94.35 × 10^5 ^Ω·m, a standard value for bulk platinum. The ground electrode is placed on the photoreceptor (subretinal) side and axially shifted by 15 mm away from the stimulation disc electrode. The ground was defined as a 100 mm diameter platinum disc electrode.

The retina was modelled as an inhomogeneous element with a piecewise linear change in resistivity, from 35 Ω·m at the photoreceptor layer to 2 Ω·m in the RGC layer. These values were extrapolated from measurements carried out in macaques, *in vivo *[22]. Even though mammalian eyes exhibit differences in sizes, thickness of retinal layers (including the nerve fibre layer), etc. which are all critical factors under consideration, the reason for the extrapolation is that the anatomical organisation of the primate retina closely resembles that of humans [23]. The selection for thickness of the retina depends mainly on the location of an epiretinal implant. Typically, in retinal implantation trials [24], the implant is placed closer to the fovea, but not over it [25] due to the absence of ganglion and bipolar cells in the fovea. The retinal thickness in the region surrounding the fovea is known to vary between ~100 μm at the foveal floor to ~320 μm at the foveal rim [26]. Considering that the location of the implant near the fovea is not precise, the chosen value for the retinal thickness was 200 μm.

**Figure 1:**
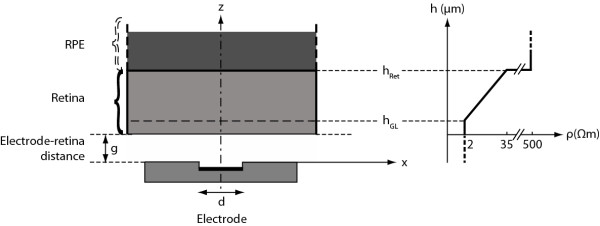
**Schematic representation of the various elements of the simulation framework**. A schematic representation of the elements constituting the simulation framework (excluding the ground electrode) and a graphic representation of the resistivity change as a function of the retina depth. RPE is the retinal pigment epithelium, *h_GL _*is the depth at which ganglion cells are assumed to be located, *h_Ret _*is the depth where the retina ends and the RPE starts, *g *is the electrode-retina distance and *d *is the electrode disc diameter.

Two geometrical factors affecting the activation thresholds were included in the simulation model as variable parameters, electrode-retina distance (*g*) and electrode disc diameter (*d*). The retinal resistivity model is positioned according to the electrode-retina distance, between 0 μm and 1500 μm. The diameter of disc electrodes was defined to be 260 μm and 520 μm to mimic the electrode geometry used in the human trials [13]. Other variables defined in the model are: *h_GL _*- depth at which ganglion cells are assumed to be located, and *h_Ret _*- depth where the retina ends and the RPE starts. *h_GL _*was defined to be 20 μm outwards from the epiretinal side, i.e., (*g*+20) μm from the surface of the implant. Figure 1 presents a schematic representation of the above mentioned elements (excluding the ground electrode) and the variable parameters together with a graph representing the resistivity change as a function of the retina depth.

### Threshold current and depth of RGC activation

The threshold current necessary for activation of an RGC by means of extracellular stimulation has been both experimentally and theoretically demonstrated to depend upon various parameters such as activation of soma versus axon (axon initial segment) [27-29], stimulus pulse type (cathodic or anodic), polarity (monophasic or biphasic) and shape (pulse duration) [20,30]. For the purpose of our study, we consider a spherical RGC soma (without axon and dendrites) activated using a single, balanced, cathodic pulse of 0.975 ms (per phase) duration at threshold excitation. The rationale behind choosing a spherical model of an RGC soma instead of planar (disc-like) or cylindrical (unmyelinated axon-like) was based on a recent modelling study by Boinagrov *et al*. [20] employing the six-channel salamander RGC model [31]. They calculated strength-duration curves based on this model (Figure twelve, Pg. 2245 in their paper) and demonstrated good matching with experimental data [32] that were generated using large electrode (125 μm and 500 μm in diameter) stimulation. Similar range of sizes was used for electrodes in our study as mentioned in the next section. The stimulus pulse parameters were taken from Argus I clinical trials in order to be relevant for comparison with results from our study. The influence of pulse type and duration on RGC activation was neglected from our simulation framework as this was accounted for directly in the assumption for activation criterion (explained below).

An RGC activation threshold criterion can be extracted from one of the multiple strength-duration curves computed by Boinagrov *et al*. [20] using a planar Hodgkin-Huxley (HH) cell model studied using a single, charge-balanced, cathodic-first, biphasic stimulus (type used in Argus I trials). The threshold current injected to create a voltage gradient to activate an RGC located at a distance from the electrode (cell activation depth) leads to a local electric field near the cell. In the current study, an electric field criterion of 1 kV/m is chosen, assuming uniform electric field around the cell. This value corresponds to a local voltage drop (transcellular) of 10 mV for a biphasic 1 ms stimulus pulse duration and a planar Hodgkin-Huxley cell with a cell polarisation time RC of 10^-4 ^ms [20] (refer Figure five (A) in their paper). The model also demonstrates that biphasic stimulation thresholds for planar cells are lower than those of a spherical cell by a factor of 1.7-1.8 throughout all pulse durations. In spite of this factor, we considered a transcellular potential of 10 mV created across an RGC soma of around 10 μm in diameter, a typical RGC size in primates used in modelling studies previously [33]. By neglecting the factor and considering soma to be spherical, a compromise between experimental (or clinical) and modelling inaccuracies was made. By selecting another threshold electric field criterion (here, based on the strength-duration curve) would simply change the computed threshold voltages, which affects related parameters (e.g. threshold currents, electric field distribution, etc.). RGCs are considered to produce robust responses when directly activated [20,34]. Therefore, it was assumed in our studies that the retina can be directly stimulated at the *h_GL _*layer.

### FEM simulation framework

Comsol Multiphysics^® ^4.0a was used as the finite element modelling environment. An external bounding box of 44 × 25 mm drawn from the axis of the stimulation electrode is used to limit the computation space. A 3D finite element model of the stimulation and the ground electrodes was created with a mesh resolution between 0.5 M and 2 M nodes, depending on the framework configuration. A Delaunay advancing front type triangulation meshing algorithm of Lagrange-quadratic element type was utilised in Comsol for meshing the simulation volume. Element refinement (high density meshing) was performed on the stimulation and the ground electrodes to ensure current conservation. The data extracted from the simulations were post-processed to generate the required plots. The current delivered by the electrode was computed by a boundary integration of the normal component of current density over the ground electrode. Impedance is computed as the ratio between the applied voltage stimulus and the resulting current seen at the electrode taking into consideration the retina with or without an electrode-retina gap.

The time-varying bio-electric fields, currents and voltages in a biological medium can be examined in the conventional quasi-static limit [35]. In our recent modelling studies (submitted for publication elsewhere), we computed the threshold current and the impedance using both harmonic and DC modes of representing the various subdomains in our finite element simulation framework. The results suggest that the quasi-static formulation could be reduced to a simple DC model at large stimulation voltages and pulse widths for the purpose of our studies. It was observed that, at large voltage stimulus amplitudes, the voltage drop across the electrode interface impedance is relatively small. In addition, the stimulation parameters such as stimulus pulse shape and duration mainly affect only the capacitive component of the retinal tissue impedance. It was seen that the capacitive component of the tissue impedance at frequencies ranging from 1 kHz up to 10 kHz (range of stimulation pulse frequencies) is more than an order of magnitude higher than the resistive component. Consequently, a frequency independent DC mode of computation was used in the simulation framework considering retina as purely resistive along with the neglected electrode interface impedance.

**Figure 2:**
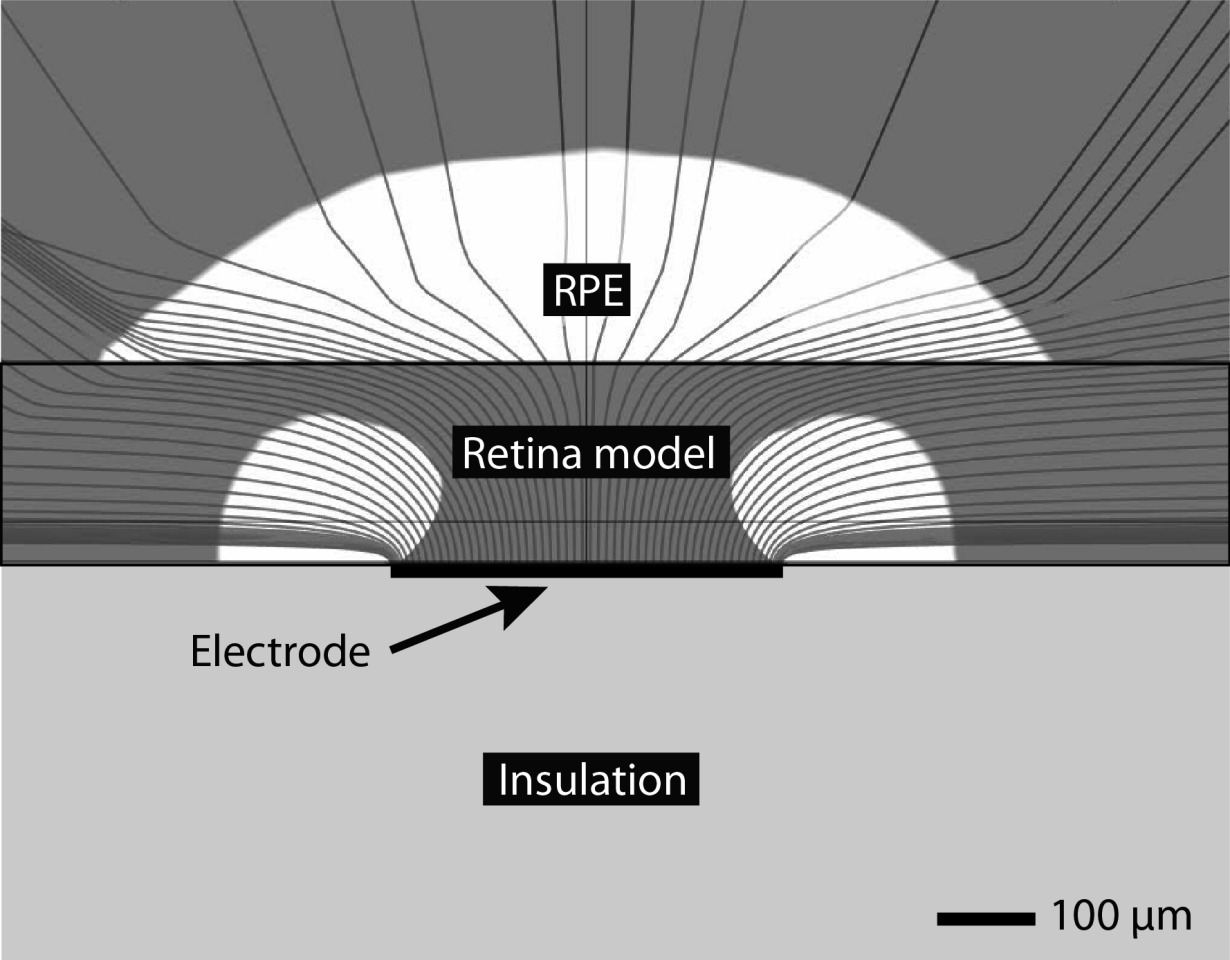
**A graphical representation of the activation area for a 400 μm diameter electrode when in contact with the retina**. A graphical representation of the electric current lines elicited by the stimulation of a 400 μm diameter electrode when in contact with the retina. The area of activation for a threshold criterion of ≥1000 V/m is represented by the white regions in the retina and RPE. The stimulation voltage was 1 V giving rise to a current of 17 μA. The threshold stimulation current was found to be ~8 μA. Note: An over-stimulation of the retina was intentionally shown here to clearly illustrate the area of activation.

Based on an earlier explanation, we emphasise that in our simulation framework, injecting a current that will produce an electric field of 1 kV/m at *h_GL _*is a sufficient condition to activate the RGCs. The FEM simulations used a monopolar stimulation scheme for which the return electrode is located in the far field. Simulations were performed with a potential difference applied between the stimulation and ground electrodes. The area of activation for a 400 μm electrode is graphically illustrated in Figure 2. The FEM was solved using a geometric multigrid iterative solver. The underlying equations employed in our simulations along with symbol notations are presented in Table 1.

**Table 1:** Equations employed in the simulation framework operated in DC

Domain/Boundary name	Type of condition	Equation (s)
• Physiological fluid	Current conservation	∇ · *J *= 0
• Retina model		*J *= *σE*
• Substrate insulation		*E *= -∇*V*

Bounding box	Electric insulation	-**n **· *J *= 0

Stimulation electrode	Electric potential	*V *= *V_stimulation_*

Ground electrode	Ground	*V *= 0

Equation to compute electric scalar potential, *V *in the medium due to an electrode stimulation		∇ · [*σ *∇*V*] = 0

Notations: *J *- current density on the electrode, *E *- electric field vector,

*V_stimulation _*- amplitude of the voltage stimulus, *σ *- conductivity of the physiological medium,

**n **- normal vector.

## Results and Discussion

The effectiveness of this computational study can be evaluated by directly comparing clinical and electrophysiological results with outcomes based on our simulation framework. One of the principal results for comparing our study are the only exhaustive measurements made during the clinical study conducted by de Balthasar *et al*. [13] on human beings implanted by Argus I epiretinal implants. The scattered impedance and threshold data observed in their experimental study was associated with small movements of the electrode array. In order to determine a theoretical water window for electrodes used in Argus I experimental protocol, charge and charge density were calculated with a stimulus duration of 0.975 ms.

### Stimulation thresholds as a function of electrode-retina distance

**Figure 3:**
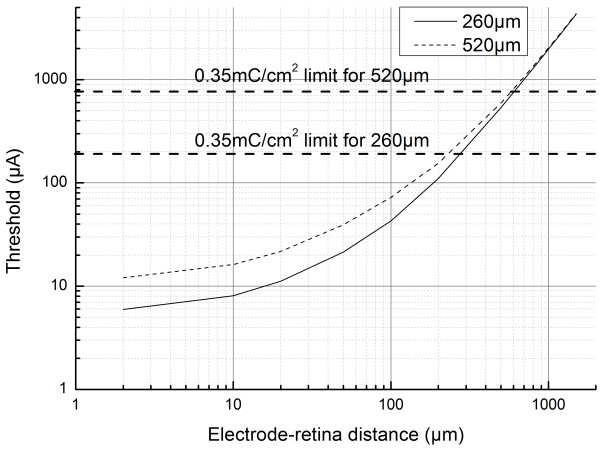
**Threshold current versus electrode-retina distance**. Evolution of computed threshold current with variation in electrode-retina distance for two electrode sizes (260 μm and 520 μm). We observe that there is a factor two difference of threshold between the two sizes when the electrode is in contact with the retina. Approximately, an order of magnitude increase in threshold current is observed when the electrode-retina distance reaches half of the electrode diameter. When electrode-retina distance exceeds the electrode diameter, the threshold current becomes proportional to the square of the distance. The corresponding charge injection limit (for 0.975 ms pulses) is displayed for both electrode sizes.

A computed threshold current is plotted as a function of electrode-retina distances for the two electrode sizes: 260 μm and 520 μm are presented in Figure 3. A factor two difference between the thresholds for both electrodes was noticed when the electrodes were in contact with the retina. We observed an approximate order of magnitude increase in thresholds when the electrode-retina distance reached half of the electrode diameter. Subsequently, for electrode-retina distances exceeding the electrode diameter, the threshold current becomes proportional to the square of the electrode-retina distance. For smaller distances (<20 μm), the threshold changes were less pronounced as also observed in *in vitro *experiments conducted by Jensen *et al*. [14]. At large electrode-retina distances, above 300 μm, the two electrodes are not differentiated as they showed nearly the same threshold current values. Threshold current variation as a function of electrode-retina distance obtained in this study, shown in Figure 3, are within the range of values obtained in the experimental results of the Argus I clinical trials [13].

Safe stimulation is critical for a chronic usage of a retinal prosthesis. Platinum electrodes have a charge density limit ranging between 0.05 [36] and 0.49 mC/cm^2 ^[37] per stimulation pulse above which electrochemical reactions dominates at the electrode surface [37]. The range of charge densities (also known as reversible charge storage capacity) is related to considerations on real surface area, geometry of the electrode and on the stimulus pulse width [38]. A theoretical charge density limit of 0.35 mC/cm^2 ^was chosen for our study considering a real geometry of the electrode and a pulse width of 0.975 ms. Currents corresponding to this charge limit are 190.6 μA for 260 μm and 762.4 μA for 520 μm electrodes. It can be observed that the current injection limit can be reached at an electrode-retina distance of about 270 μm for 260 μm diameter electrodes and nearly 600 μm for 520 μm diameter electrodes. Close proximity of RGCs to the electrodes is thus a critical issue for safe and chronic retinal stimulation.

### Stimulation thresholds as a function of electrode sizes

**Figure 4:**
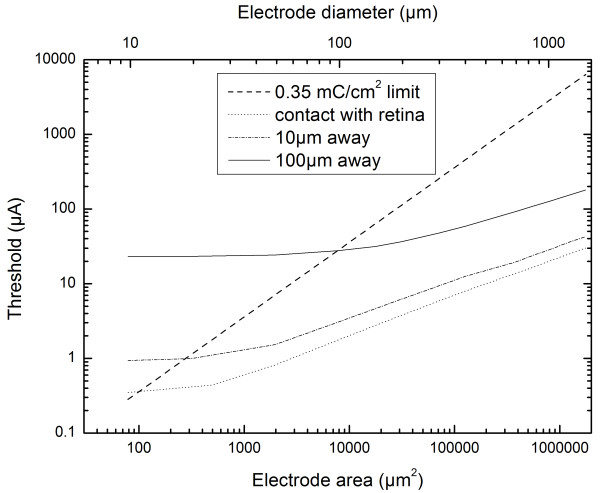
**Threshold current versus electrode diameter/area**. Computed trend for variation of threshold currents with changing electrode area (corresponding electrode diameter is shown on the top axis). Dotted line represents the charge density limit calculated for platinum electrodes using a stimulation pulse width of 0.975 ms. When electrodes are in contact with the retina, the threshold current is a power function of the square root of the electrode area (or a power law with the electrode circumference). When electrodes are not in contact with the retina, the threshold is almost independent of the electrode size until the electrode diameter is roughly equal to the electrode-retina distance, and then follows the power law. This behaviour is explained by dominance of edge effects at small electrode-retina distances. The current injection limit trend line is also plotted on the graph. It is observed that for an electrode with a radius smaller than the electrode-retina distance will typically require a stimulation current larger than the injection limit.

A range of disc electrodes with diameters ranging between 10 μm and 1500 μm were used to simulate the relationship between the stimulation threshold and electrode-retina distances - 0 μm (in contact with retina), 10 μm and 100 μm. In retina contact condition presented in Figure 4 (axes plotted in logarithmic scale), it is observed that the threshold current is a power function of the square root of the electrode area (follows a power law with the electrode circumference) as inferred from the linearity between the quantities. The charge density increases to a high value with smaller electrodes, as the decrease in surface area outweighs the threshold current decrease, explaining the change in slope of the linear trend below electrode sizes of 25 μm. Our simulation results for large electrodes (> 100 μm) are in agreement with the trends observed in *in vitro *experiments conducted by Jensen [32] and the literature review by Sekirnjak [16] groups indicating that the threshold current necessary to elicit spikes within RGCs varies as a power law with electrode area. The thresholds obtained for smaller electrodes (< 25 μm) cannot be compared with a previous report by Sekirnjak *et al*. [16] as the stimulus pulse widths used in their study was different. When the electrode is not in contact with the retina, the threshold is almost independent of the electrode size for all electrode-retina distances below a distance approximately equal to the electrode diameter and for distances above this, follows the power law again. This behaviour is explained by the electrode edge effect dominating at small electrode-retina distances. Another interesting result in relation to safe stimulation is that for an electrode with a radius smaller than the electrode-retina distance will typically require a stimulation current above its current injection capacity (refer Figure 4).

Computed threshold currents for 260 μm and 520 μm electrodes differ only slightly at electrode-retina distances from ~200 μm onwards. This similarity between threshold currents for the two electrodes was also observed in Argus I clinical trials [13]. It is interesting to notice from their measurements (threshold versus electrode-retina distance, refer Fig. seven (b) in [13]) that the similarity in thresholds for the two electrodes, results from the electrode-retina distances being in the range of 150-300 μm. The consistency offered by our predictions in comparison to the existing clinical measurements on thresholds correlating to electrode-retina distance reiterates the importance of a realistic framework.

Electrode-retina distance influences the threshold current values for various electrode sizes having a pronounced effect on safe stimulation of the retina. A trend line between threshold current limits for different disc electrodes based on the electrochemical limit for platinum (0.35 mC/cm^2^) is plotted in Figure 4. The approximate electrode sizes below which the electrochemical limit (for platinum electrodes) is exceeded for the three electrode-distance conditions is as follows: (i) 11 μm diameter when the electrode is in contact with retina, (ii) about 20 μm diameter when the electrode is within 10 μm distance from the retina and (iii) about 100 μm when the electrode is within 100 μm distance from the retina. Since both charge and charge density are to be considered for discussion on safe stimulation [38], the stimulus pulse duration is critical. Our simulation framework is capable of computing threshold currents for different electrode geometries based on a stimulus pulse dependent threshold criterion, rendering it a powerful prediction tool.

### Impedance variation based on electrode-retina distance

Impedance changes in neuroprostheses (e.g., cochlear implants) have been correlated with changes in the tissue resistivity surrounding the electrode [39] and electrochemical changes at the electrode surface with time [40]. There has been no strong evidence for these phenomena in chronic epiretinal implantation studies [13]. Moreover, the probability of an immune response (e.g. tissue encapsulation) in such implantations is low because the electrodes were observed to be in the vitreous significantly away from the retina during trials [41,42]. Consequently, by neglecting effects influencing impedance changes, impedance measurements can be compared to the simulated values for obtaining information on distance of the retina with respect to electrode array of the implant. As threshold currents reduce with closer proximity between the retina and electrodes, impedance can be used to predict threshold currents for retinal stimulation. Studies [13,42] based on frequent monitoring of impedance during the post implantation period suggest that there is a continuous change in distance between the electrode array and the retina influencing the variation in measured impedance.

**Figure 5:**
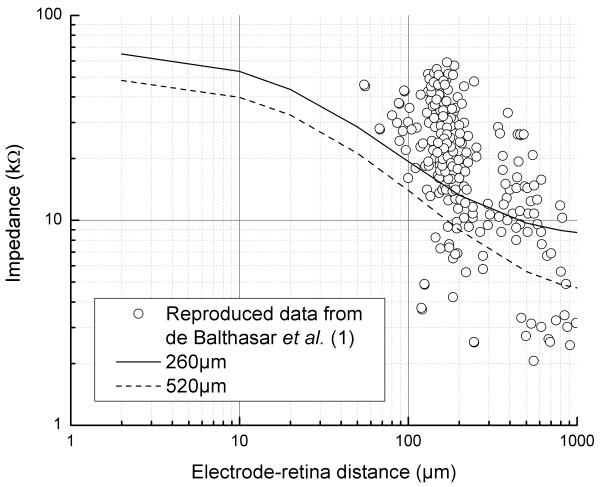
**Impedance versus electrode-retina distance**. Computed impedance change with variation in electrode distance from the retinal surface. The impedance during electrode-retina contact is not indicated. Impedance values for the contact condition for 260 μm electrode: 70.5 kΩ; 520 μm electrode: 52 kΩ. Open circles are experimental data points from the Argus I clinical trials [13]. The clinical data demonstrate large scattering of impedance but are grossly within the range of simulated values from our framework. A fitting of the experimental data is not completely relevant, but it would lead to an impedance-distance relationship that is not in contradiction with our simulations.

Our framework computed the trend between impedance and electrode-retina distance and is shown in Figure 5. By using this trend, the threshold currents can then be directly predicted from computed impedance values knowing the relationship between threshold currents and electrode-retina distance (refer Figure 3). Higher impedances (electrodes closer to the retinal surface) means low thresholds for the activation of RGCs. Electrode-retina distances which affect the computed values of impedance indicate that there is no benefit of using a smaller electrode other than the capacity to place more electrodes within the same area; as at large electrode-retina distances (especially in the range 100-300 μm), there is small difference in thresholds for different electrode sizes. But, when multiple such electrodes are stimulated simultaneously, a higher resolution might be produced as shifting stimulation of an array of four small electrodes (for e.g., half the size of larger electrode) by one row could shift the stimulation by a smaller distance than shifting stimulation of larger electrodes by one row. Even though there is a large variability within the impedance measurements presented in Argus I clinical trials [13] (reproduced in Figure 5 for convenience), they are grossly within our simulated range of values for impedance versus electrode-retina distance. Considering the data spread of impedance-distance measurements in the Argus I clinical data; a fitting of the data is not totally relevant, but a fit would not be in contradiction with our simulations.

### Estimation of resolution based on spatial extent of stimulation

**Figure 6:**
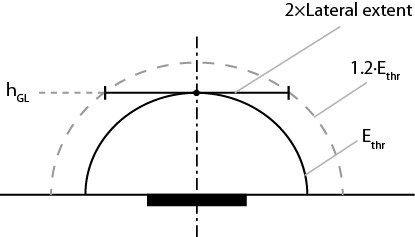
**A graphical representation of lateral extent of retinal stimulation**. An illustration of the definition for lateral extent of retinal stimulation. It is denoted by a horizontal distance measured at *h_GL _*where the threshold electric field criterion is reached for a 20% increase in stimulation amplitude. The dark block represents the stimulating electrode.

**Figure 7:**
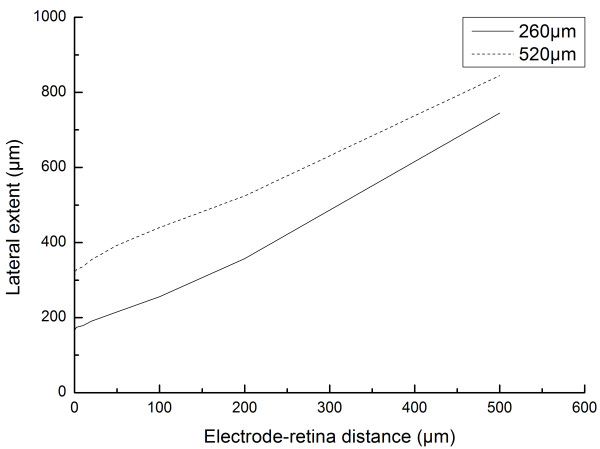
**Lateral extent of stimulation versus electrode-retina distance**. Relationship between the computed lateral extent of stimulation and the electrode-retina distance demonstrates that an increase in electrode-retina distances decreases the resolution of the retinal implant. The lateral extent is proportional to the sum of half of electrode-retina distance and radius of the electrode. For a point source (or very small electrodes), the graph would cross the origin of the graph.

In our study, we have computed threshold currents for activation of a single RGC located at the cell activation depth (*h_GL_*) from the stimulation electrode. During actual experiments, to ensure stimulation, there is a tendency to use stimulation currents 10-20% above the pre-determined minimum threshold current. In our study, we define lateral extent as the horizontal distance (measured from the electrode axis) covered at *h_GL _*corresponding to a threshold electric field of 1 kV/m caused by a 20% excess on the threshold current. An illustration of the assumption and the lateral extent definition is presented in Figure 6. Implant resolution can be calculated based on the lateral extent of stimulation for the electrodes. A relationship between the lateral extents of stimulation zone with varying electrode-retina distances for both the electrodes has been plotted in Figure 7. The lateral extent is proportional to the sum between half of the electrode-retina distance and the radius of the electrode. The lateral extent for a point source electrode (or very small electrodes) would be zero ideally. The linear-like relationship between the lateral extents of stimulation and the electrode-retina distance implies that the resolution of the implant drops with increasing electrode-retina distances for the electrode geometries studied.

## Conclusions

Simulations on the effect of geometrical factors, viz. electrode size and electrode distance to the retinal surface affecting impedance and threshold values is an indication of the importance of proximity between the electrode array and the retina for a successful retinal implant. Resolution of the implant can be estimated for different electrode-retina distances considering the computed lateral extents of stimulation. Electrode breakdown and tissue encapsulation effects, in spite of being extremely important in the viability of neural prostheses, have not been observed to be dominant in implanted human retinal prostheses studied until now. This could be due to the fact that they have not been studied long enough to evaluate their performance under long term exposure to retinal milieus. Based on the threshold and impedance data collected during clinical epiretinal trials conducted in human subjects until now [13,41], the variation in threshold current and impedance can be linked to changes in electrode-retina distance (Pg. 281, Chapter 14 of [43]). Hence, the pre-experimental computation of characteristic dependency between threshold and impedance is generally a significant guideline and supplemental information for surgeons and electrophysiologists. Furthermore, the presented simulation framework is a powerful and useful tool for implants' designers - as it can be used to predict threshold of each electrode, irrespective of its geometry, in arrays of high electrode count targeted at high-resolution retinal stimulation in future.

An integrated simulation framework computing electric fields in the electrode-retina interface could help in understanding the effective operation of a retinal implant. Knowledge of current densities in the retinal tissue can resolve significant questions which include: design of implantable electrode arrays, a proper location for the implant to be placed, optimal electrode geometry and ground placement, efficiency of different shapes and sizes of electrodes, optimal inter-electrode spacing, maximum amount of current injected safely for a given configuration, efficiency of current injection and current circulation in a tissue for a particular scenario.

## Competing interests

Gregoire Cosendai is affiliated with Second Sight Medical Products. The other authors declare that they have no proprietary, financial, professional, or other personal competing interests of any nature or kind.

## Authors' contributions

HK and PR designed the model and simulations. HK performed all computations and simulations. HK and WH wrote the manuscript together. WH along with HK was involved in systematic organisation and representation of results. GC and AB supplied critically important intellectual content in revising the manuscript. All authors read and approved the final manuscript.
